# Clinical evaluation of ultra-high-field MRI for three-dimensional visualisation of tumour size in uveal melanoma patients, with direct relevance to treatment planning

**DOI:** 10.1007/s10334-016-0529-4

**Published:** 2016-02-25

**Authors:** Jan-Willem M. Beenakker, Teresa A. Ferreira, Karina P. Soemarwoto, Stijn W. Genders, Wouter M. Teeuwisse, Andrew G. Webb, Gregorius P. M. Luyten

**Affiliations:** Department of Radiology, C.J. Gorter Center for High-field MRI, Leiden University Medical Center, P.O. Box 9600, 2300 RC Leiden, The Netherlands; Department of Ophthalmology, Leiden University Medical Center, P.O. Box 9600, 2300 RC Leiden, The Netherlands; Department of Radiology, Leiden University Medical Center, P.O. Box 9600, 2300 RC Leiden, The Netherlands

**Keywords:** Magnetic resonance imaging (MRI), Uveal melanoma, Ophthalmology, Eye, Ultrasound

## Abstract

**Objectives:**

To assess the tumour dimensions in uveal melanoma patients using 7-T ocular MRI and compare these values with conventional ultrasound imaging to provide improved information for treatment options.

**Materials and methods:**

Ten uveal melanoma patients were examined on a 7-T MRI system using a custom-built eye coil and dedicated 3D scan sequences to minimise eye-motion-induced image artefacts. The maximum tumour prominence was estimated from the three-dimensional images and compared with the standard clinical evaluation from 2D ultrasound images.

**Results:**

The MRI protocols resulted in high-resolution motion-free images of the eye in which the tumour and surrounding tissues could clearly be discriminated. For eight of the ten patients the MR images showed a slightly different value of tumour prominence (average 1.0 mm difference) compared to the ultrasound measurements, which can be attributed to the oblique cuts through the tumour made by the ultrasound. For two of these patients the more accurate results from the MR images changed the treatment plan, with the smaller tumour dimensions making them eligible for eye-preserving therapy.

**Conclusion:**

High-field ocular MRI can yield a more accurate measurement of the tumour dimensions than conventional ultrasound, which can result in significant changes in the prescribed treatment.

## Introduction

The advantages of ultra-high-field human MRI compared to lower clinical fields primarily revolve around the increased signal-to-noise ratio (SNR), which can be used to increase the spatial resolution in a given imaging time. One of the aims of translating ultra-high-field MRI into true clinical applications is to determine which types of application require a higher spatial resolution than is currently achieved at clinical field strengths of 1.5 and 3 T. The major challenges include the increase in tissue *T*_1_ relaxation times and decreased *T*_1_-tissue contrast, the (in general) decreased *T*_2_ relaxation times, and the increased sensitivity to differences in magnetic susceptibility. This means that clinical scanning protocols from lower fields must be re-optimised in terms of the different relaxation times. A recent paper provided a comprehensive overview of the current clinical applications of ultra-high-field MRI [1].

One topic that was not specifically covered in this particular review was ophthalmic MRI. The potential advantages for clinical use revolve directly around the increased spatial resolution achievable within acceptable scan times (since short scans must be used to minimise eye-motion-induced image artefacts): this increased spatial resolution is key for identifying small pathologies within the eye. In recent years, MRI has become a promising new diagnostic imaging modality in ophthalmology, a discipline that relies mainly on optical imaging techniques [2–9]. However, eye motion results in a high degree of image artefacts, which interfere with the diagnosis. By employing dedicated scan paradigms such as cued blinking [10], high-resolution, essentially motion-free MR images of the eye can be obtained. Compared to the conventional optical measurement techniques prevalent in ophthalmology departments, MRI has two key advantages that allow it to provide additional valuable clinical information. First, unlike optical imaging methods, MRI is not affected by refraction, which can lead to systematic geometrical measurement errors. For this reason, ocular MRI is being used by many different groups to generate geometrical eye models to study ocular conditions such as myopia [5, 11–14]. Second, the ability of MRI to image opaque tissues such as ocular tumours and optic nerve pathologies offers the potential to improve the diagnosis of patients suffering from these diseases [3, 6, 9, 15].

Uveal melanoma is the most common primary intraocular malignant tumour in adults [16–20]. In the past, enucleation (surgical removal of the eye) was the main treatment, but in the last 10 years various eye- and vision-saving treatments have become available, including ruthenium plaque brachytherapy and proton beam radiation therapy [21]. The optimal treatment is mainly determined by the location and size of the tumour. Ruthenium plaque brachytherapy, for example, spares most of the healthy surrounding tissue, resulting in optimal preservation of visual function, but is only suitable for small to medium sized tumours that are not proximate to the optic nerve. For larger tumours external beam therapy can be prescribed, but in a number of cases the eye needs to be removed.

**Fig. 1:**
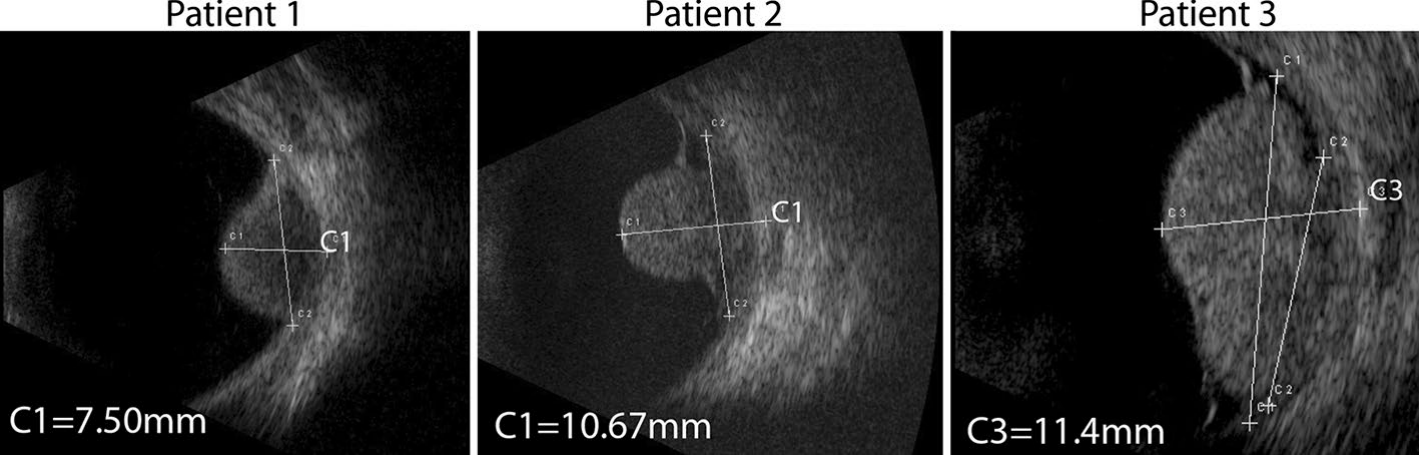
B-scan ultrasonographic images of the eyes of three patients showing uveal melanoma

For a patient to be eligible for ruthenium plaque brachytherapy the maximum distance between the outside of the sclera, where the plaque is located, and the tumour is 7 mm [22]. Clinically, the extent of the tumour is primarily determined using 10 MHz B-scan ultrasonography, as shown in Fig. 1, in which an ultrasound transducer is manually positioned to acquire a two-dimensional image. To measure the prominence of the tumour correctly, the transducer needs to be positioned perpendicular to the tumour, which is often not possible because of the surrounding tissue, e.g. the nose. These oblique cuts through the tumour result in an over-estimation of the tumour size, with the degree of over-estimation being patient/tumour specific, meaning that no simple correction algorithm can be applied. Furthermore, since ultrasound only measures two-dimensional cross sections of the tumour, it can potentially miss the location of the maximum tumour size. Recent advances in ocular MRI make it possible to image the complete tumour in three dimensions, potentially allowing a much better determination of the maximum tumour prominence. In this article, we show the MR data acquired in ten patients with uveal melanoma using a custom-built RF coil at 7 T and compare the tumour dimensions determined from the MR images with those acquired via clinical ultrasound evaluation.

## Materials and methods

The patients were admitted to the Department of Ophthalmology at the LUMC, and all data were acquired according to the Declaration of Helsinki. After the standard clinical evaluations (optical and ultrasound measurements), an additional 7-T ocular MRI examination was performed. For the ultrasound a probe (Quantel Medical Aviso, Cournon d’Auvergne France) was used with a transducer frequency of 10 MHz, an exploration angle of 50°, focus of 24–26 mm, axial resolution of 200 µm and lateral resolution of 600 µm (data supplied by the manufacturer). Ultrasound images from three representative patients are shown in Fig. 1.

**Fig. 2:**
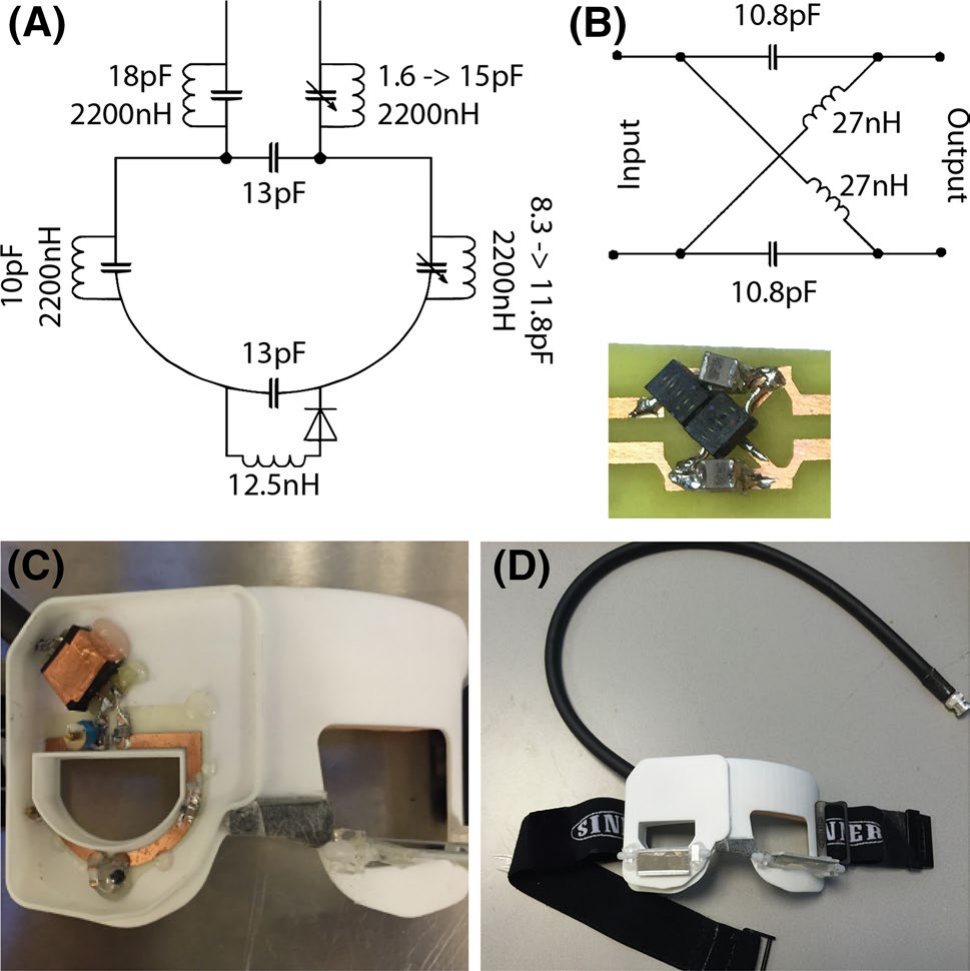
Schematics and pictures of the single-channel receive eye coil. The dimensions of the resonator are 45 × 35 mm^2^. **a** Values of lumped elements used to tune and impedance match the coil at 298.1 MHz. **b** To decouple the cable from the resonator an LC-Balun was used because of its stability and small size. **c** Photograph of the coil plus LC-Balun on the 3D printed former. **d** Mirrors were attached to the coil housing to allow the patient to look at a screen positioned at the end of the magnet bore

The MRI measurements were performed on a Philips (Best, The Netherlands) Achieva 7-T whole-body magnet [9]. A custom-made dedicated receive eye coil was used in combination with a volume transmit coil (Nova Medical Inc., Wilmington, MA, USA). In previous research scans a three-channel eye-coil array was used: in this design the conductive wires of the resonator blocked the view of the eye. In the coil used in this study, a single-channel receive coil was constructed as this allowed the patient to look through the centre of the coil: furthermore the robustness of the coil was improved by construction on a printed circuit board and the use of a miniature LC-Balun. Details of the coil design are shown in Fig. 2.

To reduce susceptibility artefacts, the affected eye was closed with a piece of tape and covered with a piece of wet gauze [23]. The patients were instructed to focus with the non-affected eye on a Malthusian cross during the scans. As the movement of the imaged eye is highly correlated with movement of the non-imaged eye, eye blinking of the non-imaged eye results in severe motion artefacts. These eye-motion artefacts were minimised by the use of a cued-blinking protocol consisting of a regular break every 3 s, in which the scanner was automatically paused and the subjects were visually instructed to blink [10].

MR images were acquired using a 3D inversion recovery turbo gradient echo technique (MP-RAGE) with an inversion time of 1280 ms, a shot interval of 3 s, and a turbo field echo factor of 92. The TR/TE/tip angle was: 6.7 ms/3.4 ms/16°. The scan time was 3 min and resulted in a spatial resolution of 0.4 × 0.4 × 0.9 mm^3^ and a field of view (FOV) of 40 × 46 × 38 mm^3^. Two orthogonal reconstructions were made from this scan, from which additional two-dimensional scans were planned with different types of contrast. A turbo spin-echo sequence was used to acquire four slices of 1 mm thickness with a TR of 2500 ms and an echo train length of 10. The scan was performed both with and without spectral inversion recovery (SPIR) fat suppression and had an in-plane resolution of 0.3 × 0.3 mm^2^. The slice gap was adjusted for every patient in order to cover the complete tumour, and the scan took slightly more than 1 min. Then a 3D spoiled gradient echo with a TR/TE/tip angle of 10 ms/2.9 ms/6°, a spatial resolution of 0.5 × 0.5 × 1.0 mm^3^ and an FOV of 40 × 46 × 38 mm^3^ was acquired in 1 min and 16 s. Finally a multi-slice inversion recovery with SPIR fat suppression was acquired with an echo-train length of 16, a resolution of 0.3 × 03 mm^2^ and a total acquisition time of 3.5 min.

## Results

**Fig. 3:**
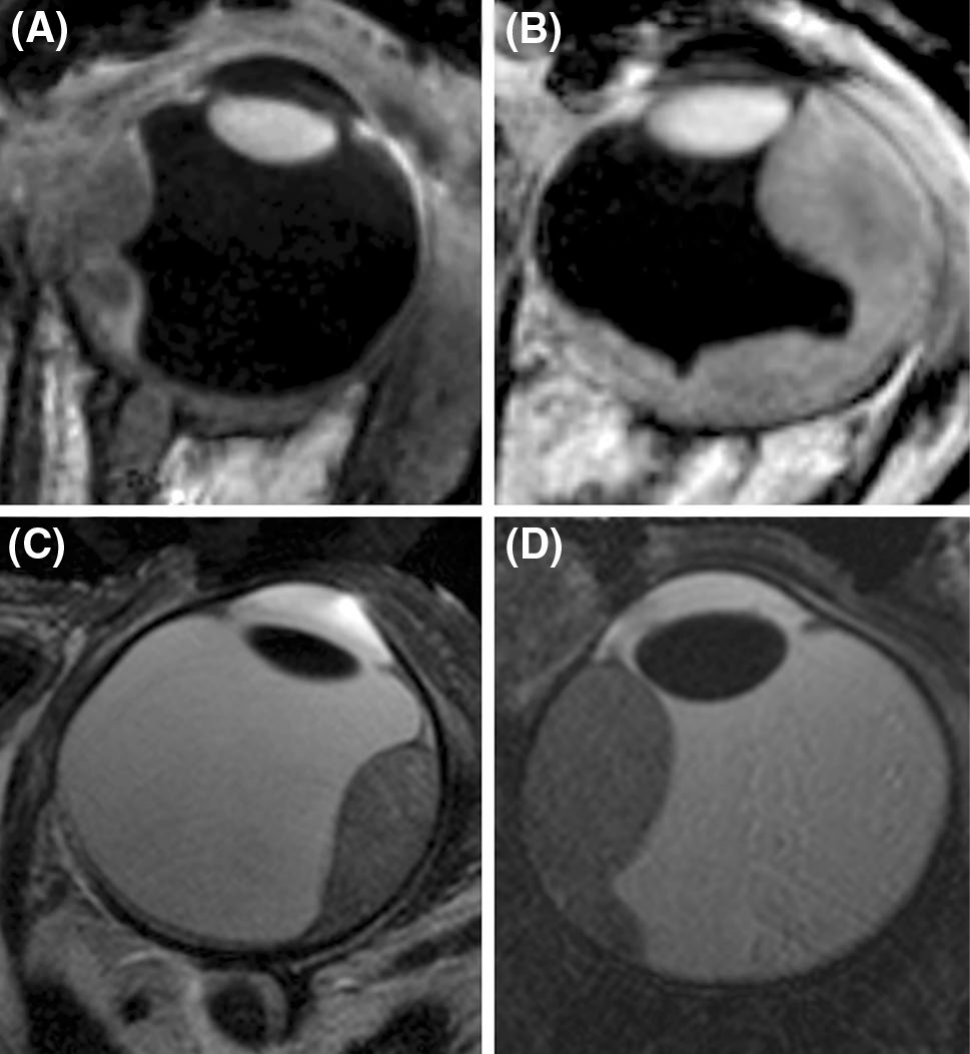
High-resolution MR images from four patients (the images of three additional patients are shown in Fig. 4). The images illustrate the wide variety of tumour shapes and locations in UM. **a**, **b** Images acquired with the 3D MPRAGE sequence, which provides high contrast between the tumour and vitreous body. The 3D nature of these images allows for an accurate assessment of the tumour dimensions. **c**, **d** Fat-suppressed 2D spin-echo images show a hypointense sclera, allowing to screen for extra-scleral tumour extensions

**Fig. 4:**
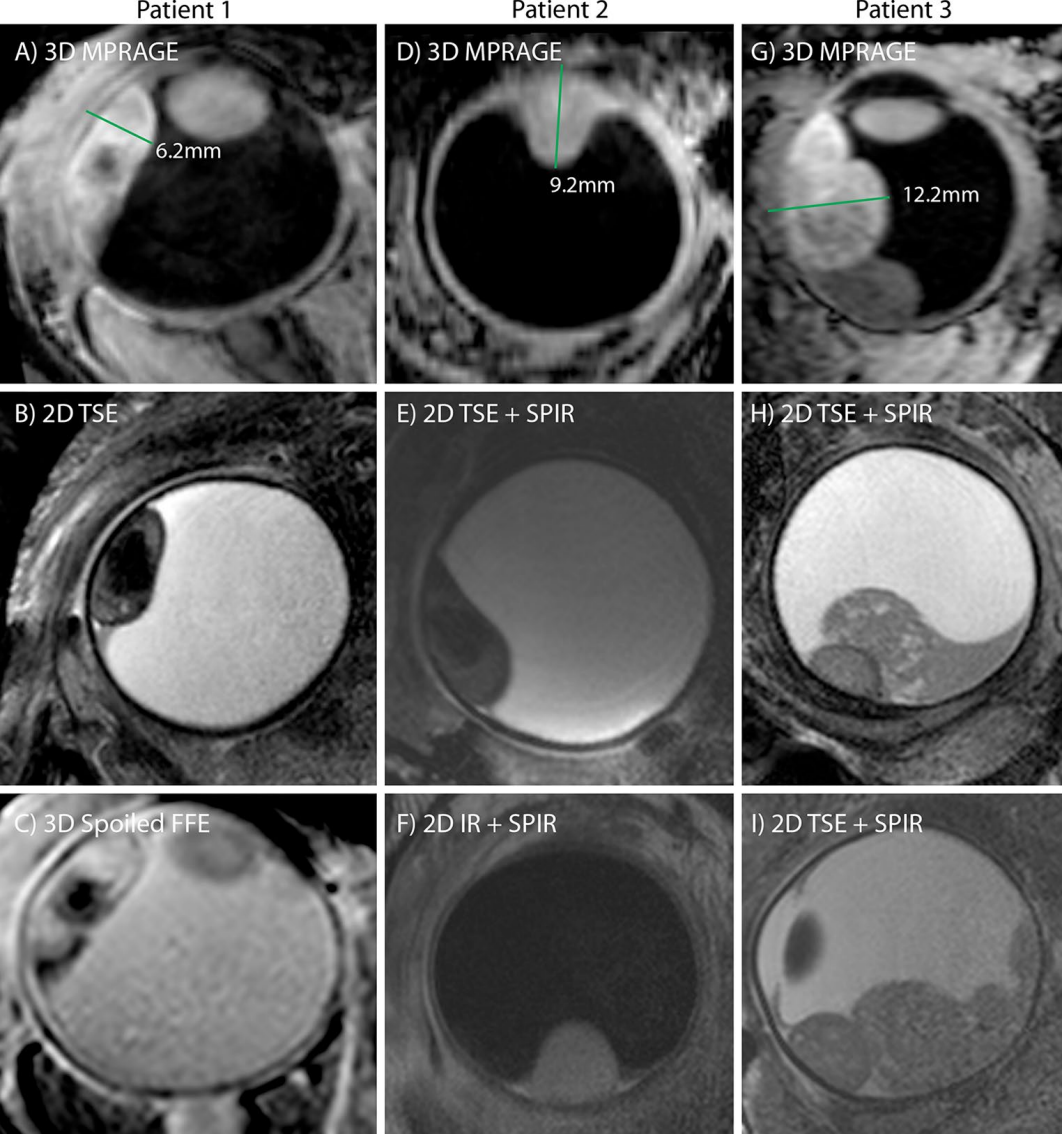
High-resolution images from the three patients from Fig. 1 [patient 1 (**a**, **b**, **c**), patient 2 (**d**, **e**, **f**) and patient 3 (**g**, **h**, **i**)]. The tumour prominence (*green line*) was measured on the images acquired with the 3D MPRAGE sequence (**a**, **d**, **g**). (**b**, **e**, **h**, **i**) 2D spin-echo acquisitions measured perpendicular to the tumour. **c** A 3D spoiled gradient FFE shows a hypointense structure within the tumour. **i** A fat-suppressed inversion recovery sequence can be used to screen for tumour infiltrations through the sclera

For all patients the MRI scans resulted in motion-free images in which the tumour could clearly be delineated, representative images are shown in Figs. 3 and 4. The inversion recovery based scans (Figs. 3a, b and 4a, d, g) show the highest contrast between the tumour and the surrounding vitreous body, and the sclera can be differentiated from the uveal layer. The three-dimensional nature of this scan allows a complete assessment of the spatial extent of the tumour. These images, however, show a low contrast between the sclera and orbital fat (it should be noted that the ultrasound images in Fig. 1 show an even poorer contrast between these tissues). For the assessment of extra-scleral extensions of the tumour, however, a better delineation of the sclera is needed since the boundary between the surrounding hypo- and hyperintense layers can be very convoluted. To this end, the high-resolution *T*_2_-weighted multi-slice spin-echo images, shown in Figs. 3d, e and 4b, e, f, h, i, were acquired, which clearly show the hypointense sclera. Finally, Fig. 4c shows the images from the 3D spoiled gradient echo sequence. Although this sequence yields a lower SNR than the other sequence types, it does have high intra-tumour contrast. The hypointense region in the centre of the tumour of Fig. 4a–c is speculated to be a necrotic region rich in melanin. Since the eye of this patient did not need to be enucleated, this hypothesis could (fortunately) not be verified by histology.

**Fig. 5:**
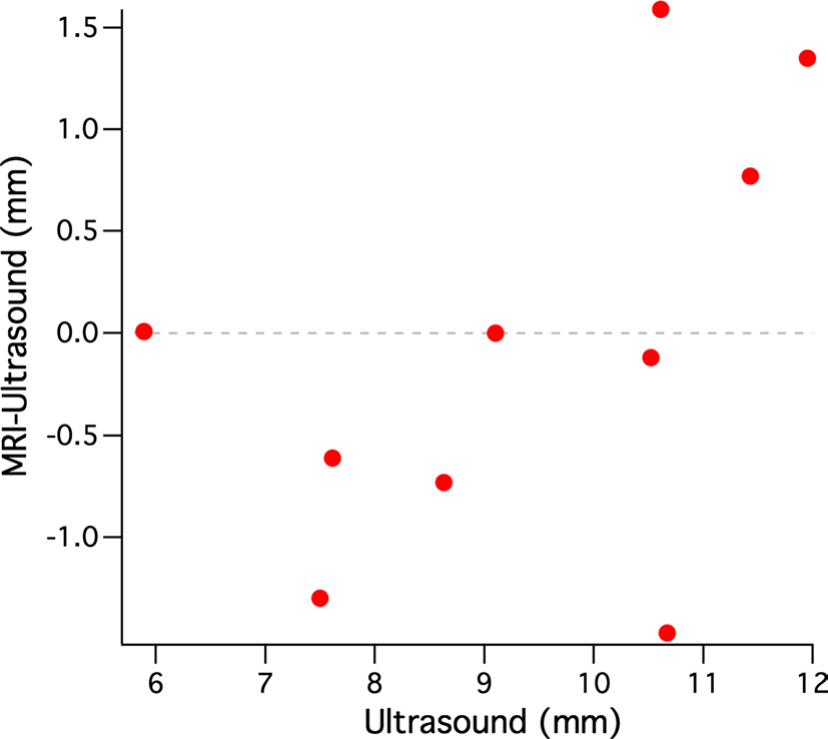
Comparison between the ultrasound and MRI measurements of the tumour prominence of ten uveal melanoma patients. In 5 of these patients the 3D MRI evaluation revealed a smaller tumour compared to the 2D ultrasound images. In the three patients with a large and complexly shaped tumour, the MRI showed a larger tumour prominence

The maximal prominence of the tumour for each patient was assessed on the 3D inversion recovery scan using a thee-dimensional viewer. Figure 4 shows the result of this assessment for the three representative patients from Fig. 1. For patient 1 the MR images showed a prominence of 6.2 mm, for patient 2 a prominence of 9.2 mm and for patient 3 a prominence of 12.2 mm. The results for all patients are summarised in Fig. 5. For two patients the MRI showed the same tumour prominence as the ultrasound. For five patients, including patients 1 and 2 from Figs. 1 and 3, the MRI revealed a smaller tumour prominence compared to the ultrasound. In three cases, including patient 3, the MR images showed a larger tumour. The tumours of these patients were relatively large (>10 mm) and consisted of multiple lobes.

## Discussion

For 80 % of the patients the MRI-based 3D evaluation of the tumour prominence resulted in a different tumour prominence compared to the ultrasound. For some of the large tumours, which consisted of multiple lobes, the MRI showed an up to 1.6 mm larger tumour. As the eyes of these patients were not eligible for eye-preserving therapy, this did not have any influence on the proposed treatment. In five of the ten patients, however, the MRI revealed an up to 1.5 mm smaller tumour. For two of these patients (including patient 1 from Figs. 1 and 3), this resulted in a substantial change in treatment plan. For these patients the original ultrasound measurements showed a tumour with a prominence slightly too large for ruthenium plaque therapy, which for this patient would result in the enucleation of the eye. The MRI, however, showed a slightly smaller tumour that would still be eligible for ruthenium plaque therapy, which meant that the eye could be spared. Because of the uncertainties of the ultrasound, especially the potential oblique cuts through the tumour, the final decision was based on the MR images, and ruthenium plaque therapy was offered. (The total radiation dose delivered by a ruthenium plaque decreases as the distance from the plaque increases; therefore, an accurate determination of the maximum distance between the outer sclera and tumour is needed to select which patients are eligible for this treatment.)

As small millimetre-size differences in tumour prominence can result in significant changes in the treatment, accurate length measurements are essential to assess the optimal treatment. Although higher field MRI enables an increased spatial resolution, magnetic susceptibility effects are greater, which could potentially distort the images. The application of wet gauze to the imaged eye effectively shifts the air/tissue interface much further away from the eye. Small air bubbles can sometimes be present below the eyelid and can produce very local distortions in the image, but as the tumours are located posteriorly the image of the tumour itself is not affected. Further evidence that high-field MRI gives accurate ocular length measurements was provided in previous work that showed a high degree of agreement, <1 % difference, compared to the gold standard of optical biometry [5]. Overall, all of these considerations indicated a high accuracy of MRI-based geometrical measurements in the eye. Furthermore, as the MR images provide a higher contrast between the sclera and orbital fat than ultrasound, MRI is able to provide a better delineation of the tumour boundaries, justifying an MRI-based determination of the optimal treatment.

To assess the reproducibility of the measurements, both the ultrasound and MR images were retrospectively evaluated by two additional independent observers. The MRI showed a slightly lower difference between the measurements compared to the ultrasound, an average SD of 0.22 and 0.32 mm respectively. Clinically, the variability of the ultrasound evaluation will be significantly higher as the measurement is very dependent on the orientation of the ultrasound transducer. A comprehensive study by Char et al. [24] showed a 0.60-mm interobserver variability in the ultrasound evaluation. As the MRI acquires a full 3D description of the tumour, it provides the possibility to make a cross section in every direction, removing this significant source of variability. Furthermore, the MRI allows for a retrospective verification of the orientation in which the measurement was taken, which cannot be done with the 2D ultrasound.

In addition to the described diagnostic value of the additional MRI scans, the evaluation of these patients revealed two other potential benefits of MRI compared to ultrasound. First, the full three-dimensional data on the tumour geometry can be used for more precise planning for the different forms of external beam radiotherapy of uveal melanoma: this is currently based on two-dimensional fundus photography and ultrasound images. Second, the high intra-tumour contrast compared to ultrasound potentially enables a better determination of the tumour response to therapy. Currently, only changes in the outer tumour dimensions can be monitored, which are known to change after the full course of therapy is finished. The higher contrast of MRI could show tumour response at an earlier stage, enabling a more patient-specific treatment.

## Conclusion

This work has shown the diagnostic potential of ultra-high-field ocular MRI for uveal melanoma. In eight of the ten patients presented, the three-dimensional MR images provided a better measurement of the tumour dimensions compared to conventional ultrasound. For two of these patients this resulted in a significant change in treatment. The ultrasound suggested the tumour was too large for ruthenium plaque brachytherapy, in which case removal of the eye would have been considered. The three-dimensional MRI data, however, enabled a more precise measurement of the tumour dimensions, resulting in a slightly lower tumour prominence, which is still feasible for brachytherapy. This resulted in the preservation of these particular patient’s eye.
